# Case Report: Exome sequencing reveals recurrent *RETSAT *mutations and a loss-of-function *POLDIP2 *mutation in a rare undifferentiated tongue sarcoma

**DOI:** 10.12688/f1000research.14383.1

**Published:** 2018-04-26

**Authors:** Jason Y. K. Chan, Peony Hiu Yan Poon, Yong Zhang, Cherrie W. K. Ng, Wen Ying Piao, Meng Ma, Kevin Y. Yip, Amy B. W. Chan, Vivian Wai Yan Lui

**Affiliations:** 1 Department of Otorhinolaryngology, Head and Neck Surgery, Faculty of Medicine, The Chinese University of Hong Kong, Hong Kong, Hong Kong; 2 School of Biomedical Sciences, Faculty of Medicine, The Chinese University of Hong Kong, Hong Kong, Hong Kong; 3 Department of Computer Science and Engineering, Faculty of Engineering, The Chinese University of Hong Kong, Hong Kong, Hong Kong; 4 Chongqing Medical University, Chongqing, China; 5 Department of Anatomical and Cellular Pathology, Faculty of Medicine, The Chinese University of Hong Kong, Hong Kong, Hong Kong

**Keywords:** Head and neck, Sarcoma of the tongue, low mutational burden, POLDIP2 and RETSAT mutations

## Abstract

Soft tissue sarcoma of the tongue represents a very rare head and neck cancer with connective tissue features, and the genetics underlying this rare cancer are largely unknown. There are less than 20 cases reported in the literature thus far. Here, we reported the first whole-exome characterization (>×200 depth) of an undifferentiated sarcoma of the tongue in a 31-year-old male. Even with a very good sequencing depth, only 19 nonsynonymous mutations were found, indicating a relatively low mutation rate of this rare cancer (lower than that of human papillomavirus (HPV)-positive head and neck cancer). Yet, among the few genes that are somatically mutated in this HPV-negative undifferentiated tongue sarcoma, a noticeable deleterious frameshift mutation (with a very high allele frequency of >93%) of a gene for DNA replication and repair, namely *POLDIP2 *(DNA polymerase delta interacting protein 2), and two recurrent mutations of the adipogenesis and adipocyte differentiation gene *RETSAT* (retinol saturase), were identified. Thus, somatic events likely affecting adipogenesis and differentiation, as well as potential stem mutations to *POLDIP2*, may be implicated in the formation of this rare cancer. This identified somatic whole-exome sequencing profile appears to be distinct from that of other reported adult sarcomas from The Cancer Genome Atlas, suggesting a potential unique genetic profile for this rare sarcoma of the tongue. Interestingly, this low somatic mutation rate is unexpectedly found to be accompanied by multiple tumor protein p53 and *NOTCH1* germline mutations of the patient’s blood DNA. This may explain the very early age of onset of head and neck cancer, with likely hereditary predisposition. Our findings are, to our knowledge, the first to reveal a unique genetic profile of this very rare undifferentiated sarcoma of the tongue.

## Introduction

Soft tissue sarcomas of the head and neck are an uncommon heterogeneous group of malignancies ^1^, with the most common subtype being malignant fibrous histiocytoma ^2, 3^. Yet, undifferentiated sarcoma of the tongue represents an even rarer tumor within this heterogeneous group of sarcomas with limited treatment options. Thus far in the literature, only about 20 cases have been reported ^4^. At the time of writing, the underlying genetic aberrations of this rare undifferentiated soft tissue cancer of the tongue remains unknown. Here, we describe a case report of an undifferentiated sarcoma of the tongue with associated pathological analysis and whole-exome sequencing (WES) of the tumor to identify the underlying genetic changes and attempt to determine if there exists any potential druggable mutations for treatment.

## Case report

A 31-year-old Chinese male who had a 1-pack-year smoking history, was a non-drinker, had no significant past medical history, no exposure to radiation, no family history of carcinomas, particularly no family history of members with young onset malignancies, presented in December of 2016 with an anterior tongue mass. Patient biopsy revealed it to be an undifferentiated sarcoma. Further magnetic resonance imaging (MRI) demonstrated a lesion localized to the tongue that involved the extrinsic tongue musculature. Subsequently the patient underwent a total glossectomy with bilateral selective neck dissection levels I–III with an anterolateral thigh-free-flap reconstruction in January 2017. The pathological findings from the surgical specimen are further described below. WES analyses were performed on the fresh surgically resected tumor (tumor content, >50%) and a paired blood sample. Adjuvant chemotherapy and radiation were recommended for this patient, but both options were declined because of concerns regarding long term toxicity and the effects on speech and swallowing. As of October 2017, the patient was disease-free with no documented recurrences on repeated MRI of the oral cavity and neck.

### Pathological findings

**Figure 1.**
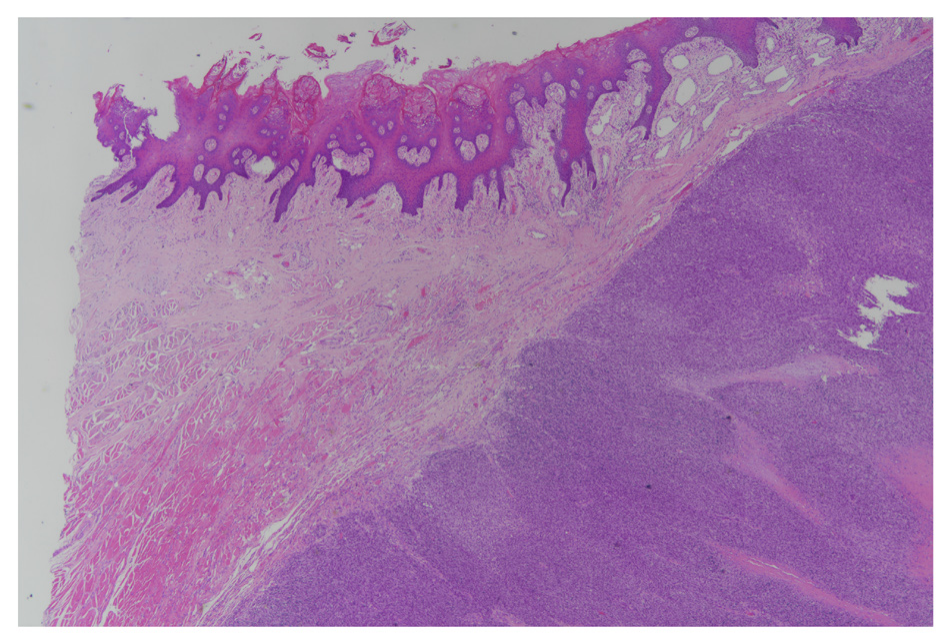
Sarcoma forming cellular fascicles is seen in the stroma of the tongue, focally invading the intrinsic muscle (left upper corner). The surface squamous epithelium shows no dysplastic field change or connection with sarcoma. (H&E stain; magnification, ×20).

The tumor mass measured grossly 7 × 7 × 5.5 cm on examination following resection. On microscopic examination, the tumor was composed of mostly spindle cells arranged in short fascicles or a vague storiform pattern. Frequent mitotic figures and prominent tumoral necrosis were seen. No osseous, chondroid or rhadomyomatous differentiation was seen on haematoxylin and eosin staining ( Figure 1– Figure 3). The closest margins were 2 mm and located at the postero-inferior, right and left margins.

**Figure 2.**
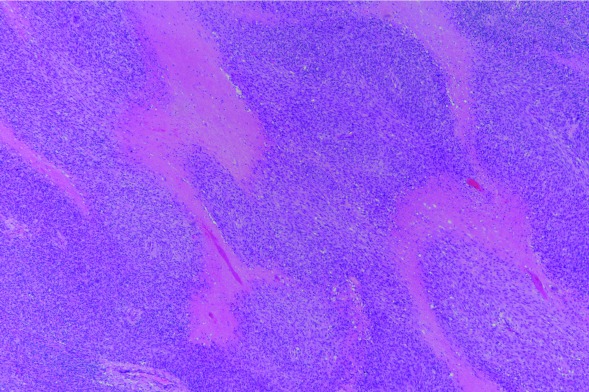
Tumor necrosis (pink foci) was prominent in the undifferentiated sarcoma. (H&E stain; magnification, ×40).

**Figure 3.**
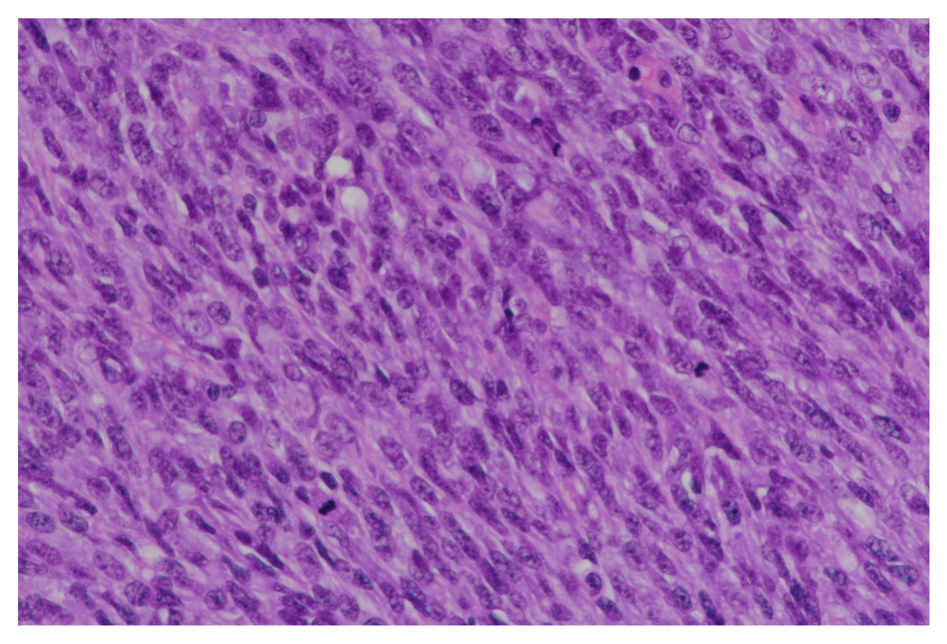
The sarcoma tumor cells possessed enlarged, elongated, pleomorphic and hyperchromatic nuclei, distinct nucleoli and small amount of cytoplasm. Mitotic figures were frequently noted. No osseous, chondroid or rhadomyomatous differentiation was seen. (H&E stain; magnification, ×400).

Further immunohistochemical studies showed that the tumor cells were positive for vimentin and p16; had focal weak positivity for SMA; had moderate positivity for TLE-1; and were negative for p63, EMA, AE1/AE3 antibodies, c-Kit, S100, CD31, CD34, desmin, myogenin, melan A, HMB45 antibody, ALK-1, human herpesvirus 8 (HHV8), CD99 (MIC-2) and SRY-box 10 (SOX10). Calponin was equivocal. No loss of integrase interactor 1 (INI1) staining was noted. PCR for high-risk human papillomavirus (HPV) DNA was negative. The Molecular Break-Apart FISH test for SS18 translocation was negative. Overall the pathological features demonstrated a high-grade sarcoma with no definite line of differentiation, consistent with an undifferentiated sarcoma.

### Mutational profile of the patient’s tumor

WES at >200x depth (Illumina HiSeq 4000) was performed to determine the genetic aberrations underlying this rare form of head and neck cancer. Normal DNA from the patient’s blood was also subjected to WES at the same depth. Surprisingly, WES revealed only 19 non-synonymous mutations in the tumor. The very low rate of non-synonymous mutations is uncommon in HPV-negative and HPV-positive head and neck squamous cell carcinoma (HNSCC) ^5^, and is slightly lower than that of reported sarcomas.

The somatically mutated genes were, in the order of allele frequencies of these 19 mutational events, *POLDIP2* (DNA polymerase delta interacting protein 2), tubulin gamma complex associated protein 3 *TUBGCP3,*, mutated in colorectal cancers ( *MCC*), *TUBA3D* (tubulin alpha 3d), *DDX11* (DEAD/H-box helicase 11), *CWF19L2* (CWF19 like 2, cell cycle control), *ZNF91* (zinc finger protein 91), *RETSAT* (retinol saturase) (2 mutations), *PRR21* (proline rich 21), *TAS2R46* (taste 2 receptor member 46), *FAM186A* (family with sequence similarity 186 member A), *HEATR5A* (HEAT repeat containing 5A), *VPS4B* (vacuolar protein sorting 4 homolog B), *PRAMEF12* (PRAME family member 12), *FAM170B* (family with sequence similarity 170 member B), *BBS4* (Bardet-Biedl syndrome 4), *ARHGAP5* (Rho GTPase activating protein 5) and *ATAD3B* (ATPase family, AAA domain containing 3B) ( Table 1). Strikingly, based on the functional annotation of these mutated genes, five of the top seven mutated genes with high allele frequencies (>10%) were known to be involved in DNA replication, and mitosis ( Table 1). This suggests that major somatic mutations of this rare tumor appear to affect DNA replication and mitosis, consistent with an aggressive phenotype. Of note, the patient’s tumor carried a 93% allele frequency of the *POLDIP2* S28fs mutation, which is a hotspot mutation in multiple cancers. In addition, the *MCC* gene was also mutated in this rare tumor ( *MCC* p.G20S) at a high allele frequency of 28%, indicating its likely role as a driver event for tumorigenesis. Interestingly, the *PRR21* mutation is also found to be mutated at high frequencies in TCGA HNSCC cohort with a hotspot mutation S86Gfs*291 and M48Tfs*329 mutation, while in this tumor, *PRR21* mutation occurred at G114C, a position near to these two hotspot mutations in HNSCC.

**Table 1.** List of the 19 non-synonymous mutations found in the undifferentiated sarcoma with their mutation type, allele frequency and potential function.

Gene	Chromosome position	Mutation type	Nucleotide change	Amino acid change	Allele Frequency	Full gene name	Potential gene function
*POLDIP2*	chr17:26,684,390	Frameshift insertion	c.83dupG	Ser28fs	93%	DNA polymerase delta interacting protein 2	DNA replication and DNA repair
*TUBGCP3*	chr13:113,201,852	Frameshift deletion	c.1235_ 1249delCGCGCGACTTTCCCA	Thr412_ Pro416del	50%	Tubulin gamma complex associated protein 3	largely unknown, tubulin gamma may be involved in mitosis
*MCC*	chr5:112824054	Missense	c.58G>A	Gly20Ser	28%	Mutated in colorectal cancers	negative regulator of cell cycle
*TUBA3D*	chr2:132237643	Missense variant & splice region variant	c.377C>T	Ala126Val	15%	Tubulin alpha 3d	tubulin family, transport, mitosis
*DDX11*	chr12:31237978	Missense	c.556C>T	Arg186Trp	14%	DEAD/H-box helicase 11	putative RNA helicases
*CWF19L2*	chr11:107325241	Missense	c.274G>A	Glu92Lys	13%	CWF19 like 2, cell cycle control	likely in cell cycle control
*ZNF91*	chr19:23544852	Missense	c.929C>A	Ala310Asp	11%	Zinc finger protein 91	unknown
*RETSAT*	chr2:85570857	Missense	c.1598C>T	Ala533Val	9%	Retinol saturase	promote adipogenesis and favors normal adipocyte differentiation
*PRR21*	chr2:240982060	Missense	c.340G>T	Gly114Cys	9%	Proline rich 21	unknown
*FAM186A*	chr12:50746486	Missense	c.4129A>C	Thr1377Pro	7%	Family with sequence similarity 186 member A	unknown
*HEATR5A*	chr14:31792790	Missense	c.3768G>A	Met1256Ile	7%	HEAT repeat containing 5A	unknown
*RETSAT*	chr2:85570849	Missense	c.1606G>A	Gly536Arg	6%	Retinol saturase	promote adipogenesis and favors normal adipocyte differentiation
*VPS4B*	chr18:61060779	Missense	c.1096C>T	Arg366Cys	6%	Vacuolar protein sorting 4 homolog B	intracellular protein trafficking
*PRAMEF12*	chr1:12835741	Missense	c.343A>G	Ile115Val	5%	PRAME family member 12	unknown
*FAM170B*	chr10:50340041	Missense	c.469G>T	Ala157Ser	5%	Family with sequence similarity 170 member B	unknown
*BBS4*	chr15:73023650	Missense	c.716T>C	Ile239Thr	5%	Bardet-Biedl syndrome 4	Bardet-Biedl syndrome gene family, associated with severe pigmentary retinopathy, obesity, polydactyly, renal malformation and cognitive disability.
*TAS2R46*	chr12:11214601	Missense	c.293T>C	Leu98Pro	4%	Taste 2 receptor member 46	Taste receptor
*ARHGAP5*	chr14:32561340	Missense	c.1465G>A	Glu489Lys	4%	Rho GTPase activating protein 5	Negatively regulates RHO GTPases in cytoskeleton changes
*ATAD3B*	chr1:1431060	Missense	c.1810T>C	Tyr604His	3%	ATPase family, AAA domain containing 3B	Interact with matrix nucleoid complexes

Most strikingly, among all the somatic mutations identified in this patient, we found that recurrent somatic events of *RETSAT* ( *RETSAT* p.A533V and p.G536R mutations), a gene known to be important for the promotion of adipogenesis and normal adipocyte differentiation. It is plausible that *RETSAT* mutations may affect adipocyte adipogenesis and differentiation, related to the sarcoma features of this rare tumor.

**Figure 4.**
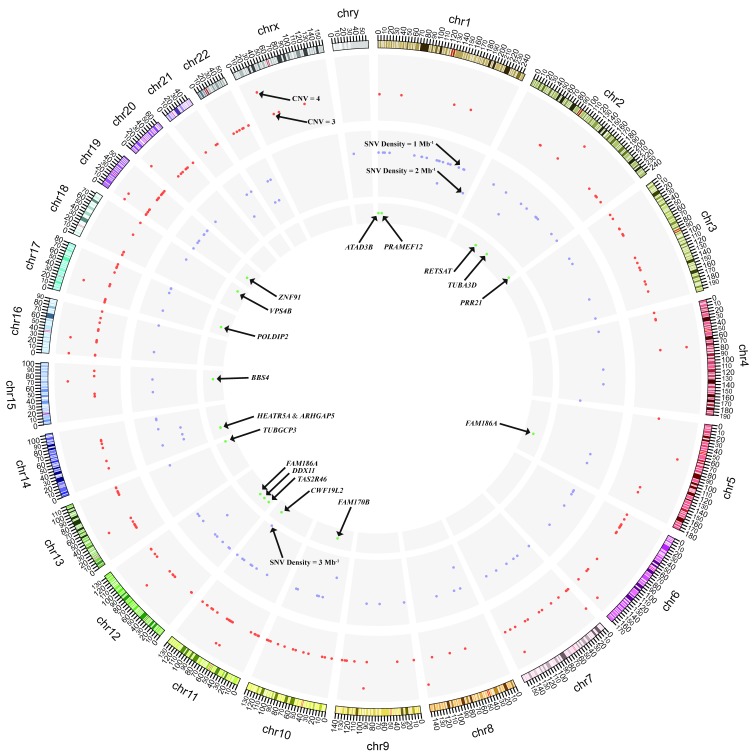
A Circos plot showing somatic genetic variations. An ideogram of a normal karyotype is shown in the outermost ring. Chromosomes are segmented into contiguous bins of 1 Mb in size. The second ring from outside shows CNVs at corresponding chromosomal positions. Distinct heights of red dots indicate different inferred copy numbers. Regions that are not covered by any dots have a copy number of two. The third ring shows SNV density. Purple dots with different heights indicate SNV densities of the corresponding regions. The innermost ring shows positions of genes with non-synonymous mutations found in the undifferentiated sarcoma. CNV, copy number variation; SNV, single nucleotide variant.

Gene copy number analysis was also performed and a total of 221 somatic copy number alterations (CNA) events were identified ( Figure 4 and Supplementary Table 1). The somatic CNA detected in this patient did not harbor any of these common HNSCC CNA events including losses of chr. 3p and 8p, as well as focal amplification or gains of chr. 3q26/28, 5p15, and 8q24 ^5^. The common CNA events in sarcomas including aberrations of the MDM2-p53 and p16-cyclin dependent kinase 4 (CDK4)-retinoblastoma-associated protein pathways, deletion of *TP53* (tumor protein p53), *CDKN2A* (cyclin dependent kinase inhibitor 2A), as well as chr. 12q13-q15 and *CDK4* amplification were also absent in this patient ^6^. These results suggest that this unique tumor is likely distinct from HNSCC and adult soft tissue sarcomas.

### Multiple germline mutations in key tumor suppressor genes

Given the young age of this patient, we also carefully examined potential aberrations in his germline mutational profile by WES of his blood DNA. Importantly, we found that this patient carried multiple germline mutational events of *TP53*, including a missense mutation of *TP53* p.P72R (100% allele frequency), 2 mutations at the immediate 5’-UTR, likely in the promoter region of *TP53* (c.-112G>A; and c.-123C>G; both with 100% allele frequencies), as well as two other intron variants with unknown effects ( Table 2). The *TP53* germline events likely explain the early age of onset for cancer for this patient. In addition, a 5’-UTR mutation, likely at the promoter region of *CDKN2A*, was identified (c.*193G>C), with an allele frequency of 100%. A *CASP8* (caspase 8) missense mutation, *CASP8* p.L14R, was also found in this patient’s blood with a 46.7% allele frequency. However, the potential function of this mutation is unclear. Lastly, this patient carries as many as 23 germline variants of *NOTCH1*; however, none of these variants are non-synonymous mutations, and the effects of these variants are largely unknown. No telomerase reverse transcriptase promoter or exon mutation was found. Lastly, no germline gene copy number alterations were observed for any critical HNSCC-associated oncogenes or suppressor genes ( Supplementary Table 2)

**Table 2.** List of germline mutations identified by gene, chromosome position, mutation type, nucleotide change, amino acid change and allele frequency.

Gene	Chromosome position	Mutation type	Nucleotide change	Amino acid change	Allele frequency
*CASP8*	Chr. 2: 202,122,995	Missense	c.41A>G	Lys14Arg	46.7
*CASP8*	Chr. 2: 202,141,512	Intron	c.838-38C>T	.	62.0
*CASP8*	Chr. 2: 202,151,163	Intron	c.1482-19A>G	.	50.4
*CASP8*	Chr. 2: 202,152,162	3_prime_UTR	c.*845T>A	.	76.9
*CDKN2A*	Chr. 9: 21,968,199	3_prime_UTR	c.*193G>C	.	100.0
*HRAS*	Chr. 11: 534,242	Synonymous	c.81T>C	His27His	44.7
*NOTCH1*	Chr. 9: 139,391,636	Synonymous	c.6555C>T	Asp2185Asp	99.4
*NOTCH1*	Chr. 9: 139,396,408	Intron	c.5473-43T>C	.	100.0
*NOTCH1*	Chr. 9: 139,396,690	Intron	c.5384+34G>A	.	100.0
*NOTCH1*	Chr. 9: 139,397,707	Synonymous	c.5094C>T	Asp1698Asp	99.4
*NOTCH1*	Chr. 9: 139,400,406	Intron	c.4015-73G>A	.	100.0
*NOTCH1*	Chr. 9: 139,400,904	Intron	c.4014+75G>A	.	100.0
*NOTCH1*	Chr. 9: 139,401,504	Intron	c.3644-79C>T	.	85.7
*NOTCH1*	Chr. 9: 139,402,663	Intron	c.3325+21A>G	.	100.0
*NOTCH1*	Chr. 9: 139,402,908	Intron	c.3172-71A>G	.	100.0
*NOTCH1*	Chr. 9: 139,403,240	Intron	c.3171+81_3171+82insC	.	100.0
*NOTCH1*	Chr. 9: 139,403,268	Intron	c.3171+54A>G	.	100.0
*NOTCH1*	Chr. 9: 139,403,554	Intron	c.2970-31A>G	.	100.0
*NOTCH1*	Chr. 9: 139,405,261	Splice_region_& intron	c.2588-4G>A	.	100.0
*NOTCH1*	Chr. 9: 139,407,452	Intron	c.2467+21G>A	.	100.0
*NOTCH1*	Chr. 9: 139,407,932	Synonymous	c.2265T>C	Asn755Asn	100.0
*NOTCH1*	Chr. 9: 139,410,177	Intron	c.1670-9A>G	.	100.0
*NOTCH1*	Chr. 9: 139,410,424	Intron	c.1669+9T>C	.	100.0
*NOTCH1*	Chr. 9: 139,410,589	Intron	c.1556-43T>C	.	100.0
*NOTCH1*	Chr. 9: 139,410,679	Intron	c.1556-133A>G	.	100.0
*NOTCH1*	Chr. 9: 139,411,714	Intron	c.1555+10A>G	.	100.0
*NOTCH1*	Chr. 9: 139,411,880	Intron	c.1442-43C>T	.	100.0
*NOTCH1*	Chr. 9: 139,412,197	Splice_region_& intron	c.1441+7C>T	.	100.0
*NOTCH1*	Chr. 9: 139,418,260	Synonymous	c.312T>C	Asn104Asn	46.2
*TP53*	Chr. 17: 7,578,115	Intron	c.672+62A>G	.	100.0
*TP53*	Chr. 17: 7,578,645	5_prime_UTR	c.-112G>A	.	100.0
*TP53*	Chr. 17: 7,579,472	Missense	c.215C>G	Pro72Arg	100.0
*TP53*	Chr. 17: 7,579,643	Intron	c.96+41_97-54delACCTGGAGGGCTGGGG	.	84.4
*TP53*	Chr. 17: 7,579,801	5_prime_UTR	c.-123C>G	.	100.0

## Discussion

The development of soft tissue sarcomas in the oral cavity and head and neck region in general are rare, with the development of an undifferentiated sarcoma in the oral cavity of an even lower likelihood ^7, 8^. A US population database analysis of head and neck sarcoma patients identified that the median age of adults affected was 55–59 years old, most commonly affecting the skin and soft tissues ^2^. This contrasts with the young age of our patient at presentation (31 years). The presence of a rare soft tissue sarcoma in a young patient provided the evidence to evaluate for germline mutations in addition to the somatic mutations, leading to the discovery of multiple germline *TP53* mutations, as well as multiple *CDKN2A*, and potentially multiple *NOTCH1* mutations of unclear functions. Notably, a *CASP8* mutation was also found in his germline. It is important to note that all these genes are known to be somatically mutated in HNSCC ^5, 9^.

Interestingly, the somatic mutational profile of this tumor was rather distinct from both HNSCC and sarcomas, with a relatively low number of non-synonymous mutations. Furthermore, unlike the genetic profile of soft tissue sarcomas, our sarcoma patient lacked the common somatic mutations in *TP53*, *PTEN* (phosphatase and tensin homolog) and *CDKN2A* ^6, 10^. Moreover, his tumor also lacked the most common somatic mutations of HNSCC, such as *TP53*, *NOTCH1*, *CKDN2A*, *PIK3CA* (phosphatidylinositol-4,5-bisphosphate 3-kinase catalytic subunit alpha) and *FAT1* (FAT atypical cadherin 1) ^5, 11, 12^. This was partly contributed to by his heavy germline mutations of some of these key tumor suppressor genes, including *TP53* and *CDKN2A*, which are commonly mutated in both sarcoma and HNSCC, usually somatically. Furthermore, his germline events also carried *CASP8* and *NOTCH1* mutations, which are commonly somatically mutated in HNSCC rather than in the germline. This difference in mutation profile in conjunction with the young age of the patient led us to consider heavy germline mutations as a possible underlying cause of this rare undifferentiated sarcoma of the tongue.

Germline mutations in *TP53* are associated with familial clustering of early onset carcinomas, including pre-menopausal breast cancer, soft tissue sarcomas, adrenal cortical carcinomas or choroid plexus carcinomas, which together are classified as Li–Fraumeni syndrome based on Chompret criteria ^13– 16^. Li–Fraumeni-like syndrome represents a subset of patients with familial clustering that do not meet the criteria for Li–Fraumeni syndrome ^15^. However, *de novo* germline mutations in *TP53* have been described, proven and are thought to be relatively common ^17, 18^. Our case highlights the importance of considering *TP53* mutations in early onset soft tissue sarcomas, even in those without familial histories, as this offers the potential to appropriately manage the patient and possibly the family.

The development of soft tissue sarcomas in an uncommon location in a young patient indicated the possibility of germline events, despite the lack of a family history of carcinomas at a young age. The early detection of these mutations can be useful in treating these patients, given the consideration that radiation wherever possible should be avoided to prevent the development of radiation-induced second malignancies ^19– 22^. Regarding the surveillance for second primary cancers, full-body MRI examinations and positron emission tomography have been recommended, as opposed to exposure to radiation with computed tomography ^19^. This case highlights the need for vigilance and consideration of possible hereditary predisposition syndromes or *de novo* germline mutations in patients with rare tumours at a young age and with atypical genetic profiles.

## Methods

### Tissue collection

Tumor tissue and blood sample were collected from the patient under written informed consent according to The Joint Chinese University of Hong Kong – New Territories East Cluster Clinical Research Ethics Committee, Hong Kong SAR (protocol number CRE-2015.396). The tumor mass was freshly frozen for DNA extraction using the QIAGEN DNeasy Blood and Tissue kit. Blood DNA was extracted from patient’s buffy coat in a similar manner. DNA samples were then quantified and quality-assessed using a bioanalyzer.

### Exome sequencing and variant calling

Genomic DNA of the patient’s tumor and buffy coat were used for WES using the Agilent SureSelect Human All Exon V5 Kit, with sequencing performed using the Illumina HiSeq 4000 platform (Macrogen, Korea) with a goal coverage of ×200 for tumor and ×100 for the blood (buffy coat) sample of the same patient.

Upon sequencing, all reads (FASTQ files) were mapped to the hg19 human reference genome assembly with Burrows-Wheeler Alignment Tool (version 0.7.12). Variant calling of single nucleotide variants (SNVs) and indels was performed using the Genome Analysis Toolkit (version v3.4.0) HaplotypeCaller pipeline. Called variants were annotated with SnpEff (version 4.3), the exome sequencing data have been deposited into European Nucleotide Archive (ENA) with accession number PRJEB25783. Somatic mutations were defined as mutations found in the tumor tissue of the patient but not in the patient’s blood. All identified recurrent mutations were confirmed in Integrative Genomics Viewer (IGV) (version 2.4) as a final check of the calling (100% accurate).

### Copy number alterations (CNAs)

CNAs were analyzed from segmented WES data using the Control-FREEC copy number and genotype caller (version 11.0) in both somatic and germline mode. In both modes, captured genomic regions were characterized using the SureSelect Human All Exon V5 bed file. Somatic CNA analyses were run by referencing tumor WES data to the paired blood WES data, whereas germline CNA analysis were run by referencing blood WES data to the hg19 human genome assembly. Identified regions were annotated with ANNOVAR (version 2017-07-17) and SnpEff. Segments with CNA were manually confirmed with an IGV check. Genetic variations were visualized using Circos (version 0.69) with “chromosomes_unites = 1000000”.

### Immunohistochemical staining

The patient’s tumor tissue was FFPE-preserved and sectioned (5–8 µm) for immunohistochemical and H&E staining. Immunohistochemical staining was performed with the following antibodies: Melan A (M7196, Dako, Denmark), HMB34 (M0634, Dako, Denmark), ALK-1 (M7195, Dako, Denmark), HHV8 (NCL-HHV8-LNA, Novo, UK), MIC-2 (M3601, Dako, Denmark), SOX-10 (ACI3099, Biocare, USA), Calp (M3556, Dako, Denmark), INI1 (612110, DB transduction, USA), Vimentin (M0725, Dako, Denmark), p16 (805-4713, Ventana, USA), SMA (M0851, Dako, Denmark), p53 (M7001, Dako, Denmark), TLE1 (401M-16, Cell Marque, USA), p63 (M7317, Dako, Denmark), EMA (MS348, Thermo, UK), AE1/3 (M3515, Dako, Denmark), c-kit (A4502, Dako, Denmark), S100 (NCL-S100p, Leica, UK), CD31 (M0823, Dako, Denmark), CD34 (NCL-L-END, Leica, UK), Desmin (M0760, Dako, Denmark) and Myogenin (M3559, Dako, Denmark). Results were examined by an experienced pathologist for the presence or absence of the target proteins. Pictures were taken under a light microscope at ×20, ×40 and ×400 magnification.

### HPV analysis

The tumor cells from the formalin-fixed, paraffin-embedded sections were isolated for DNA extraction. The DNA was subjected to PCR analysis using the consensus primers GP5+/6+, as previously documented ^23^.

## Consent

Written informed consent for publication of their clinical details and accompanying images was obtained from the patient.

## Data availability

The exome sequencing data have been deposited into European Nucleotide Archive (ENA) with accession number PRJEB25783.

## Supplementary material

**Supplementary Table 1. Somatic copy number variations obtained from DNA taken from the patient’s tumor.**

**Supplementary Table 2. Germline copy number variations obtained from DNA taken from the patient’s blood.**
