# Supplementary material for: Case Report: Exome sequencing reveals recurrent RETSAT mutations and a loss-of-function POLDIP2 mutation in a rare undifferentiated tongue sarcoma
Source: F1000Res. 2018 Apr 26;7:499. [Version 1] doi: 10.12688/f1000research.14383.1 (PMC5954348; doi:10.12688/f1000research.14383.1)
Supplement: Peer review history

# Peer review: Referee response for version 1

**DOI:** 10.5256/f1000research.15650.r34003

**Published:** 2018-07-11

**Authors:** Robin L. Jones

**Affiliations:** 1 Sarcoma Unit, Royal Marsden Hospital, London, UK

This is an informative and well written report on a very rare cancer, undifferentiated sarcoma of the tongue. The authors could consider discussing the challenges of the pathological classification of sarcomas. For instance, they reference a review by Dr Nguyen and colleagues – this review cites studies dating from the 1980s. There have been clear improvements in the classification of sarcomas over this time. Consequently, this strengthens the importance of the current study which provides detailed exome sequencing data.

 I agree with other referees that it is not surprising that the profile of an undifferentiated sarcoma is different to that of a head and neck squamous cell carcinoma.

I have read this submission. I believe that I have an appropriate level of expertise to confirm that it is of an acceptable scientific standard.

# Peer review: Referee response for version 1

**DOI:** 10.5256/f1000research.15650.r33531

**Published:** 2018-05-15

**Authors:** Marietta Tan

**Affiliations:** 1 Department of Otolaryngology-Head and Neck Surgery, Johns Hopkins University School of Medicine, Baltimore, MD, USA

Undifferentiated sarcoma of the tongue is a rare malignancy of the head and neck, and its underlying genetic alterations have not previously been investigated. In this case report, the authors performed whole-exome sequencing (WES) of a tongue sarcoma and paired blood sample of a 31-year-old male. They identified a fairly low mutation rate, with mutations in *POLDIP2*, a gene important in DNA replication and repair, and *RETSAT*, a gene involved in adipogenesis. WES analysis of the patient’s blood DNA revealed mutations in *TP53*, *CDKN2A*, *CASP8*, and *NOTCH1.*

 Overall, this is a well-written case report of a rare tumor arising in a patient with an interesting germline mutational profile. The authors should consider the following:

1. Is “undifferentiated sarcoma of the tongue” a distinct clinicopathologic entity? The introduction discusses the rarity of undifferentiated sarcomas of the tongue but then references a review of malignant fibrous histiocytoma.
2. Several times throughout the manuscript, the authors compare the mutational profile of the tongue sarcoma to that of head and neck squamous cell carcinoma. They remark that the mutational profiles of the two tumor types are distinct. I would not necessarily expect that the mutational events underlying a mesenchymal-derived tumor would be similar to an epithelial-derived tumor. Can the authors comment on why they expected that the mutational profiles might be similar?

I have read this submission. I believe that I have an appropriate level of expertise to confirm that it is of an acceptable scientific standard.

# Peer review: Referee response for version 1

**DOI:** 10.5256/f1000research.15650.r33489

**Published:** 2018-05-08

**Authors:** Tsung Lin Yang

**Affiliations:** 1 Department of Otolaryngology, National Taiwan University Hospital and College of Medicine, Taipei, Taiwan

A rare undifferentiated tongue sarcoma showing recurrent *RETSAT *mutations and a loss-of-function *POLDIP2 *mutation was presented in this case report. By showing comprehensive genetic analyses, it was interesting to identify the unique presentation of the common genes found in head and neck cancer, including TP53, CDKN2A, and NOTCH1. It is undoubtedly that the genetic profiles of this rare tongue sarcoma is different from those found in the common HNSCC because of distinct pathological background. Nonetheless, the presentations of this case different from the common sarcoma merit further confirmation. If it is really the case, it infers the possibility that the anatomical factor is presumably involved. Data related to the sarcoma of head and neck is suggested to be compared. Although this case might provide a chance to evaluate the mutual influence between the germline and somatic mutation, evidences are required to confirm whether the heavy germline mutation indeed affect the incidence of common somatic mutation found in this patient.

I have read this submission. I believe that I have an appropriate level of expertise to confirm that it is of an acceptable scientific standard.
